# Polymeric nanoparticles for dual-targeted theranostic gene delivery to hepatocellular carcinoma

**DOI:** 10.1126/sciadv.abo6406

**Published:** 2022-07-20

**Authors:** Hannah J. Vaughan, Camila G. Zamboni, Laboni F. Hassan, Nicholas P. Radant, Desmond Jacob, Ronnie C. Mease, Il Minn, Stephany Y. Tzeng, Kathleen L. Gabrielson, Pranshu Bhardwaj, Xin Guo, David Francisco, Martin G. Pomper, Jordan J. Green

**Affiliations:** ^1^ Department of Biomedical Engineering and the Institute for NanoBioTechnology, Johns Hopkins University School of Medicine, Baltimore, MD 21231, USA.; ^2^ Translational Tissue Engineering Center, Johns Hopkins University School of Medicine, Baltimore, MD 21231, USA.; ^3^ Russell H. Morgan Department of Radiology and Radiological Science, Johns Hopkins Medical Institutions, Baltimore, MD 21231, USA.; ^4^ Department of Molecular and Comparative Pathobiology, The Johns Hopkins University School of Medicine, Baltimore, MD 21205, USA.; ^5^ Department of Materials Science and Engineering and the Department of Chemical and Biomolecular Engineering, Johns Hopkins University, Baltimore, MD 21231, USA.; ^6^ Departments of Neurosurgery, Oncology, Ophthalmology, and Bloomberg~Kimmel Institute for Cancer Immunotherapy, Johns Hopkins University School of Medicine, Baltimore, MD 21231, USA.

## Abstract

Hepatocellular carcinoma (HCC) develops predominantly in the inflammatory environment of a cirrhotic liver caused by hepatitis, toxin exposure, or chronic liver disease. A targeted therapeutic approach is required to enable cancer killing without causing toxicity and liver failure. Poly(beta-amino-ester) (PBAE) nanoparticles (NPs) were used to deliver a completely CpG-free plasmid harboring mutant herpes simplex virus type 1 sr39 thymidine kinase (sr39) DNA to human HCC cells. Transfection with sr39 enables cancer cell killing with the prodrug ganciclovir and accumulation of 9-(4-^18^F-fluoro-3-hydroxymethylbutyl)guanine (^18^F-FHBG) for in vivo imaging. Targeting was achieved using a CpG-free human alpha fetoprotein (AFP) promoter (CpGf-AFP-sr39). Expression was restricted to AFP-producing HCC cells, enabling selective transfection of orthotopic HCC xenografts. CpGf-AFP-sr39 NP treatment resulted in 62% reduced tumor size, and therapeutic gene expression was detectable by positron emission tomography (PET). This systemic nanomedicine achieved tumor-specific delivery, therapy, and imaging, representing a promising platform for targeted treatment of HCC.

## Teaser

A nanoparticle platform with multiple levels of cancer-specific targeting was designed to simultaneously kill and image liver tumors.

## INTRODUCTION

Hepatocellular carcinoma (HCC) accounts for 90% of liver cancer and has an estimated overall 5-year survival rate of only 10% (*1*, *2*). In the United States, incidence of HCC is increasing, and the mortality rate is rising faster than any other leading cancer (*3*). While surgery or liver transplantation can be curative, most patients present with invasive HCC and are only eligible for palliative and locoregional treatments (*4*, *5*). Broadly cytotoxic treatments, including chemotherapy and radiation, have off-target toxicities to healthy hepatocytes, which is particularly dangerous because of the high incidence of underlying liver disease (*6*, *7*). Accordingly, there is a critical need for an effective, targeted treatment option for HCC.

In contrast to chemotherapy, gene therapies can target tumors by multiple mechanisms, including targeting of the delivery vehicle, transcriptional targeting, and molecular targeting (*8*). This has led to interest in developing nucleic acid therapies. For example, oncolytic viruses have reached late-stage clinical trials for treatment of bladder cancer, glioblastoma, and head and neck cancer (*9*). However, viral gene delivery methods have elicited concern because of risk of insertional mutagenesis and acute immunogenicity, which can be life-threatening. Viruses also have limitations of cargo capacity and manufacturing challenges, which limits scale up (*10*).

In contrast, nanoparticle (NP) delivery vehicles are typically safer but historically suffered from low transfection efficiency (*11*). Poly(beta-amino-ester)s (PBAEs) are a class of synthetic polymers that were developed to overcome barriers to intracellular gene delivery (*12*). PBAEs contain hydrolysable ester bonds in the backbone, which degrade on the order of hours and reduce the toxicity of the polymer (*13*). They also contain titratable amines that buffer the endosomal pH and facilitate endosomal escape and intracellular cargo release (*14*, *15*). Cationic PBAE polymer electrostatically complexes with anionic nucleic acids to form ~100-nm particles, which is a desirable size range for intracellular delivery and for potential accumulation in solid tumors (*16*, *17*).

Transcriptional targeting uses promoters with specific activity in target cells to restrict therapeutic gene expression (*18*, *19*). There has been great interest in investigating regulatory elements that may target exogenous gene expression selectively to tissues, including the MCK (muscle creatine kinase) promoter for skeletal muscle (*20*, *21*), SP-C (surfactant protein C) promoter for lung (*22*), and APoE/hAAT (apolipoprotein E/human alpha 1-antitryspin) for liver (*23*, *24*). While promoters highly specific to just cancer cells are relatively rare, notable examples include the promoter of telomerase (*25*), progression-elevated gene-3 (PEG-3) (*26*), and prostate-specific antigen (PSA) (*27*). Alpha fetoprotein (AFP) is the main biomarker of liver cancer, as expression is undetectable in healthy adults, yet high levels are detected in ~80% of HCC cases (*28*). AFP promoter and enhancer sequences have shown efficient and selective expression in AFP-producing HCC cells (*29*).

One hurdle to gene therapy is reducing the host immune system response to therapeutic nucleic acids. One such response is the recognition of unmethylated CpG sequences, which are found in bacterial DNA, by Toll-like receptor 9 (TLR9) expressed in plasmacytoid dendritic cells (*30*, *31*). When activated, dendritic cells release cytokines to recruit and activate natural killer cells and T cells, and this has been shown to induce inflammation and shorten the duration of gene expression (*32*, *33*). Therefore, there has been interest in developing CpG-free plasmids for gene therapy, including CpG-free backbones, genes, and promoters (*34*–*36*).

**Fig. 1.**
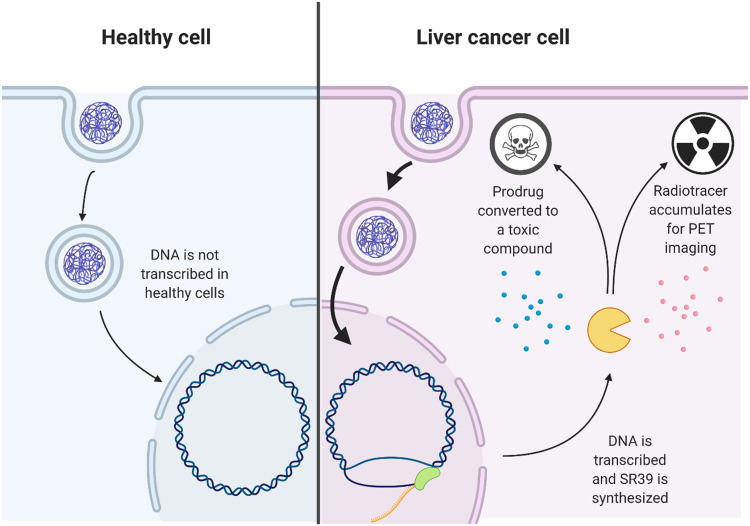
Schema describing a tumor-targeted theranostic approach for the treatment of HCC. PBAE 536 NPs are synthesized with a CpG-free transcriptionally targeted plasmid encoding for sr39. DNA delivery to healthy cells does not result in therapeutic gene expression.

Another key translational hurdle is safe and selective delivery of nucleic acids to target cancer cells. We recently reported that select PBAE formulations can enable robust and cancer-specific plasmid DNA delivery to HCC cells (*37*). However, that initial observation has not been investigated for tumor-targeted therapy or positron emission tomography (PET) imaging. Here, we develop a completely CpG-free transcriptionally targeted plasmid encoding for mutant herpes simplex virus type 1 sr39 thymidine kinase (sr39) under the control of the AFP promoter and enhancer. sr39 is a theranostic enzyme that (i) converts the prodrug ganciclovir (GCV) into a cancer-killing compound and (ii) phosphorylates radiolabeled nucleoside analogs to enable PET imaging of gene expression (Fig. 1). Combining two layers of targeting selectivity (mediated by the polymer and by the nucleic acid), we show that an HCC-specific nontoxic, biodegradable nanocarrier (PBAE 536) with a transcriptionally targeted theranostic suicide gene therapy enables sr39 delivery to HCC tumors for safe and effective tumor control and molecular genetic imaging.

## RESULTS

### Nanoparticle synthesis and characterization

**Fig. 2.**
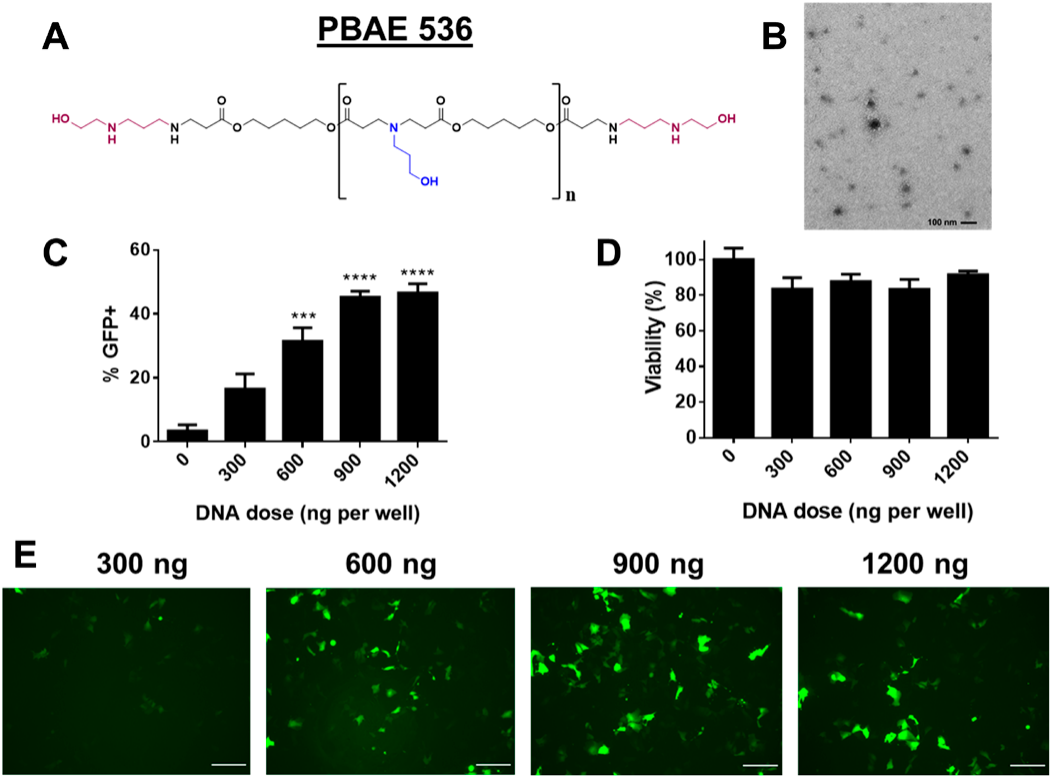
PBAE 536 NPs transfect liver cancer cells with a reporter gene in vitro. (**A**) Structure of polymer PBAE 536. (**B**) TEM image of PBAE 536 NPs. Scale bars, 100 nm. (**C**) In vitro transfection efficacy of Hep3b cells as quantified by flow cytometry. (**D**) Viability of Hep3b cells transfected with varying doses of GFP DNA. PBAE NPs were synthesized with PBAE 536 at a polymer:DNA mass ratio of 25 (w/w). Means ± SE of the mean are shown (*n* = 3). Statistically significant transfection was calculated by one-way ANOVA with Dunnett’s multiple comparisons test compared to untreated cells. (**E**) Fluorescence micrographs of GFP expression in transfected Hep3b cells. Scale bars, 500 μm. ***P < 0.001; ****P < 0.0001.

PBAE 536 (Fig. 2A) was selected for DNA delivery based on its recently found ability to preferentially transfect HCC cancer cells (*37*). The polymer was synthesized by Michael addition as previously described (*37*), with a final molecular weight of 5.64 kDa and a polydispersity index of 1.29 by gel permeation chromatography (GPC). PBAE 536 NPs harboring plasmid DNA had a hydrodynamic diameter of 137 ± 3 nm [PDI (polydispersity index) = 0.064] and zeta potential of +18.5 ± 0.6 mV. Transmission electron microscopy (TEM) images showed that dried NPs have a spherical morphology and a diameter of approximately 50 to 100 nm (Fig. 2B). Hep3b HCC cells were transfected in vitro with PBAE 536 NPs at doses of 300 to 1200 ng of green fluorescent protein (GFP) DNA per well to assess transfection efficacy. GFP expression was dose dependent with a maximal transfection rate of 47 ± 3% (Fig. 2, C and E). Viability was maintained at >80% for all formulations tested (Fig. 2D).

### Plasmid design

The HSV-TK (herpes simplex virus thymidine kinase) system is a promising approach for cancer suicide gene therapy (*38*). This enzyme catalyzes the phosphorylation of GCV, converting it into a nucleotide analog, which inhibits DNA polymerization in dividing cells (*38*). sr39 is a mutant form of HSV-TK with increased affinity for GCV (*39*). 9-(4-^18^F-fluoro-3-hydroxymethylbutyl)guanine (^18^F-FHBG) is a substrate of sr39 used for molecular genetic imaging (*40*, *41*). ^18^F-FHBG accumulates in cells transfected with sr39, and this activity may be imaged using PET to monitor therapeutic exogenous gene expression. This theranostic functionality allows for optimization of the treatment protocol, enabling precision dosing.

**Fig. 3.**
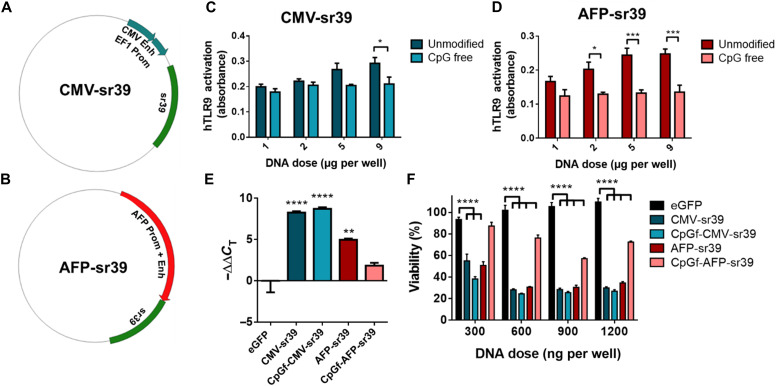
CpG-free sr39 plasmids induce sr39tk expression in vitro without elevated TLR9 activation. (**A** and **B**) Plasmid maps are shown for CMV-sr39 and AFP-sr39. (**C**) hTLR-9 activation in HEK-Blue reporter cells after exposure to CpG-containing and CpG-free CMV-sr39 and (**D**) AFP-sr39 plasmid DNA. Differences in TLR9 activation between unmodified and CpG-free plasmids were determined by two-way ANOVA and Sidak’s multiple comparisons test. (**E**) qRT-PCR analysis of sr39 expression in transfected cells. (**F**) Hep3b cellular viability 5 days after transfection with sr39 NPs and treatment with GCV (1.25 μg/ml). Differences among DNAs were determined by two-way ANOVA and Sidak’s multiple comparisons test. Means ± SE are shown for all graphs (*n* = 3). *P < 0.05; **P < 0.01; ***P < 0.001; ****P < 0.0001.

The sequence of the wild-type herpes simplex virus (type 1/strain RH2) thymidine kinase gene was modified to produce the sr39 mutant (*39*). The coding sequence for sr39 was placed under the control of a human elongation factor 1 (EF1) alpha core promoter with a mouse cytomegalovirus (CMV) enhancer sequence (Fig. 3A), enabling ubiquitous expression of the theranostic gene (CMV-sr39). For a transcriptionally targeted vector, the sr39 sequence was placed under the control of a human AFP promoter and enhancer (AFP-sr39) (Fig. 3B). Full sequences are reported in the Supplementary Materials (table S1).

CpG-free versions of these plasmids were designed to reduce TLR9 activation and reduce the risk of inflammation (*36*, *42*). In the sr39 coding sequence, codon redundancy was used to eliminate CpG sequences while maintaining the amino acids translated. Known human transcription factor binding sites within the human AFP promoter and enhancer sequences were identified using PROMO (*43*, *44*). Six CpG sequences were identified, with each sequence overlapping with a known transcriptional regulatory element by only one base pair. The nonoverlapping base pair was changed to remove the CpG, with purines exchanged for purines (G to A) and pyrimidines for pyrimidines (C to T). Final CpG-free plasmids were constructed by cloning CpG-free sr39 into a completely CpG-free pCpGfree-mcs backbone with the included CpG-free EF1-CMV promoter (CpGf-CMV-sr39) or replacing that promoter with the engineered CpG-free AFP promoter and enhancer (CpGf-AFP-sr39). Control plasmids with CpG-containing sr39 sequence and regulatory sequences were designed in a control CpG containing pCpGrich-mcs backbone. These control plasmids containing CpG are referred to as “unmodified” CMV-sr39 and AFP-sr39.

To assess the effect of removing CpG on plasmid immunogenicity, HEK-Blue reporter cells for human TLR9 activation were incubated with CpG-containing or CpG-free sr39 plasmids. Both CMV-sr39 and AFP-sr39 DNA containing CpG caused dose-dependent elevated TLR9 activation (Fig. 3, C and D). The CpG-free versions of these plasmids did not cause any detectable TLR9 activation, demonstrating that removal of CpG sequences reduces the innate immune response to these therapeutic plasmids. To evaluate the effect of CpG on promoter strength, Hep3b cells were transfected with each sr39 plasmid using PBAE 536 NPs, and quantitative reverse transcription polymerase chain reaction (qRT-PCR) was performed on isolated mRNA. Both CMV-sr39 and CpGf-CMV-sr39 promoted robust sr39 expression, with no detrimental effect from removing the CpG sequences (Fig. 3E). AFP-sr39 also showed significant expression but had a decrease in expression following removal of CpG sequences. This result highlights the importance of balancing the decrease on immunogenicity with possible decreased efficacy when using CpG-free vectors.

Despite differences in relative promoter strength, Hep3b cells transfected with each sr39 formulation with 4 days of GCV treatment showed a significant decrease in viability, with maximal effect at a DNA dose of 900 ng per well (Fig. 3F). Under these conditions, cancer cell viability was reduced to 25 ± 1% and 57 ± 1% with CpGf-CMV-sr39 and CpGf-AFP-sr39, respectively. In addition, CpGf-CMV-sr39 maintained superior cell killing effect to wild-type HSV-TK, despite extensive modifications to the coding sequence (fig. S1). The optimal concentration of GCV for in vitro cell killing was determined to be 1.25 μg/ml because of the strong therapeutic effect in sr39-transfected cells with no toxicity to cells transfected with control GFP NPs (fig. S2).

### In vitro evaluation of sr39 transcriptional targeting

**Fig. 4.**
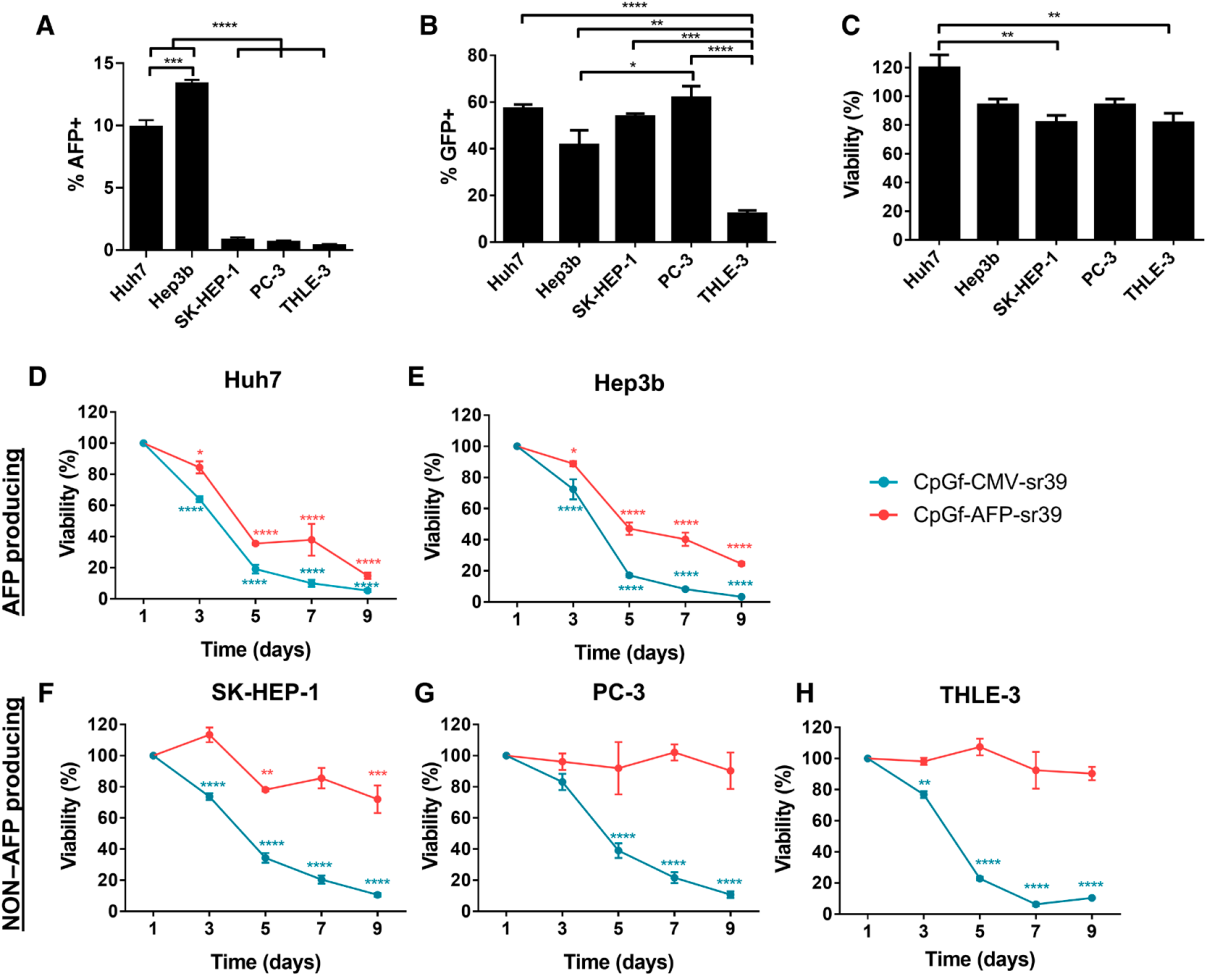
AFP transcriptional targeting restricts sr39-mediated cell death to AFP-producing HCC cells. (**A**) AFP expression in fixed and permeabilized Huh7, Hep3b, SK-HEP-1, PC-3, and THLE-3 cells measured by flow cytometry. (**B** and **C**) Transfection efficacy and viability of cell lines transfected with GFP DNA (600 ng per well) using PBAE 536 NPs. Statistically significant differences between cell lines were determined using one-way ANOVA with Tukey’s multiple comparisons test. (**D** to **H**) Viability time course of cells transfected with CpG-free CMV-sr39 and **AFP-sr39 DNA and treated with GCV (1.25 mg/ml). Loss in viability for each DNA was calculated by two-way ANOVA with Dunnett’s multiple comparisons between each time point and day 1. Means ± SE are shown for all graphs (*n* = 3). *P < 0.05; **P < 0.01; ***P < 0.001; ****P < 0.0001. n.s., not significant.

To evaluate transcriptional targeting in heterogeneous populations, several cell lines were evaluated for sr39 cell killing in vitro. Huh7 and Hep3b are AFP-producing HCC cells, SK-HEP-1 is a non–AFP-producing HCC cell line, PC-3 is a non–AFP-producing prostate cancer cell line, and THLE-3 is a normal human hepatocyte cell line (*45*). AFP expression was confirmed by flow cytometry (Fig. 4A). Each cell line was transfected with a GFP reporter gene using PBAE 536 NPs and screened for expression to determine transfection rates. All cancer cell lines showed significantly elevated transfection of 40 to 60%, compared with 12% in hepatocytes (Fig. 4B). This result was expected, as PBAE 536 NPs were optimized by screening for high transfection rates in HCC cell lines and low transfection in hepatocytes (*37*). All transfected cell lines maintained >80% viability (Fig. 4C).

Next, each cell line was transfected with CpG-free sr39 plasmids using PBAE 536 NPs, treated with GCV, and viability was measured over 9 days (Fig. 4, D to H). In all lines, cells transfected with CpGf-CMV-sr39 showed significant cell death, with 3 to 10% remaining viability by day 9. With CpGf-AFP-sr39 transfection, there was also significant cell death in AFP-producing HCC cells, with 26 ± 1% and 15 ± 2% viability on day 9 in Hep3b and Huh7 cells, respectively. However, in all non–AFP-producing cells, viability remained high for the course of the study. PC-3 and THLE-3 cells were >90% viable, while SK-HEP-1 cells’ viability dipped to 72 ± 9% by day 9, indicating that there may be limited activity in SK-HEP-1 cells despite no detectable AFP production. Specificity was further tested in a coculture of AFP-producing Huh7 HCC cells and THLE-3 healthy human hepatocytes. After 5 days, CpGf-AFP-sr39 transfection with GCV treatment decreased the proportion of AFP-producing HCC cells in culture from 10:1 to 1:1 (fig. S3).

**Fig. 5.**
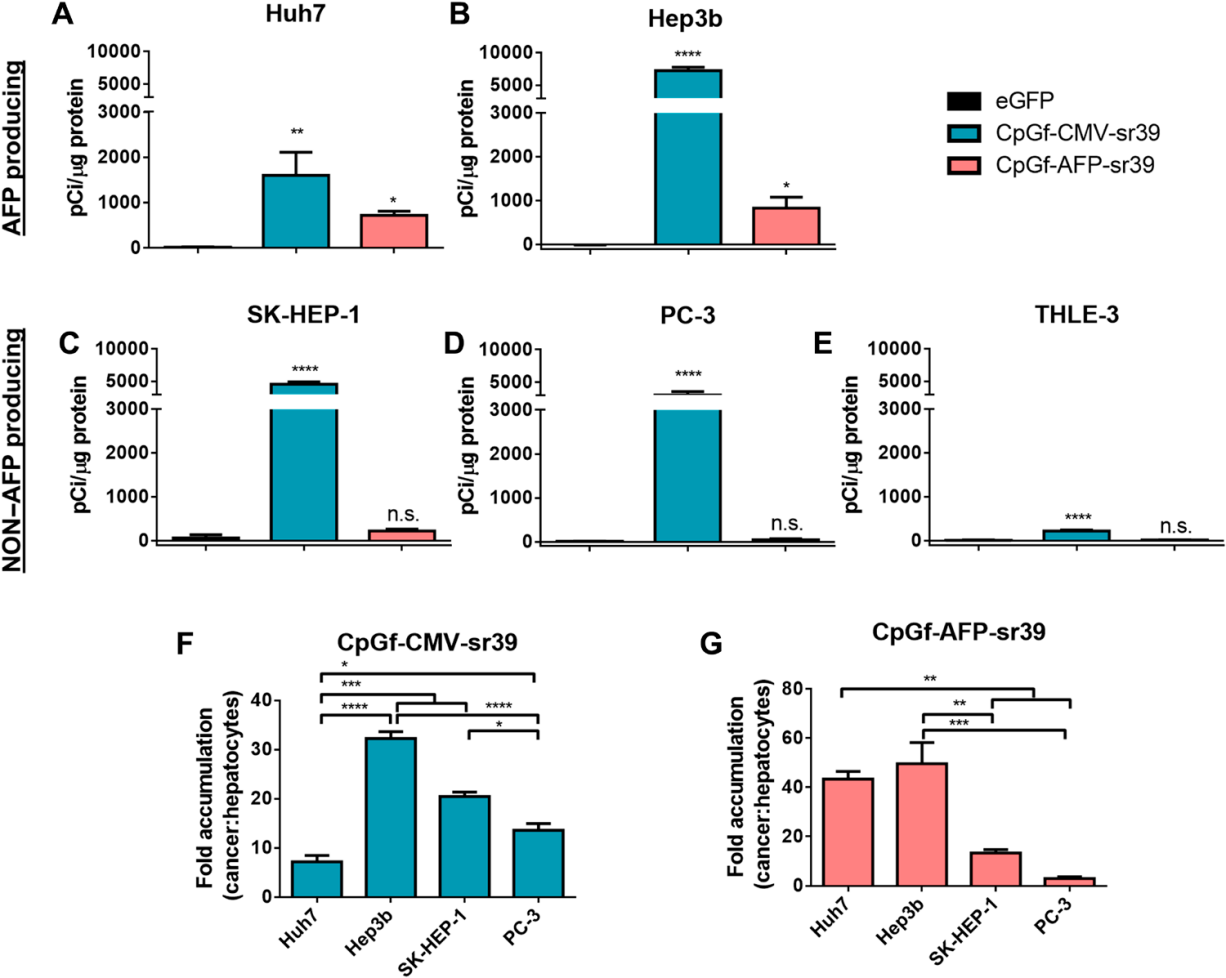
sr39 NPs enable specific accumulation of ^18^F-FHBG in target HCC cells. (**A** to **E**) Cellular radioactivity in transfected cells incubated with 10 μCi of ^18^F-FHBG for 1 hour, normalized to total protein content. Differences were calculated by one-way ANOVA with Dunnett’s multiple comparisons test between treatment groups and controls. (**F** and **G**) Fold radioactivity accumulation in cancer cells normalized to THLE-3 cells treated with the same NP. Means ± SE are shown for all graphs (*n* = 3). *P < 0.05; **P < 0.01; ***P < 0.001; ****P < 0.0001. n.s., not significant.

To evaluate the utility of this approach for molecular genetic imaging, transfected cells were incubated with 10 μCi/ml (0.37 MBq/ml) ^18^F-FHBG, and in vitro tracer uptake was measured 1 hour later (Fig. 5, A to E). Significant radioactivity was measured in all cells transfected with CpGf-CMV-sr39, ranging from 1600 to 7200 pCi/μg protein in cancer cells and 220 pCi/μg protein in THLE-3 hepatocytes. With CpGf-AFP-sr39 transfection, significant accumulation was only measured in AFP-producing HCC cells, with 800 ± 100 pCi/μg and 720 ± 50 pCi/μg protein in Hep3b and Huh7, respectively. While not statistically significant, SK-HEP-1 cells transfected with CpGf-AFP-sr39 had 220 ± 20 pCi/μg protein, again suggesting low levels of activity. Transfection with CpGf-AFP-sr39 resulted in 43 ± 3– and 50 ± 9–fold higher accumulation in Huh7 and Hep3b over THLE-3, and this HCC specificity was not observed with CpGf-CMV-sr39 (Fig. 5, F and G).

### In vivo gene delivery to orthotopic liver tumors

**Fig. 6.**
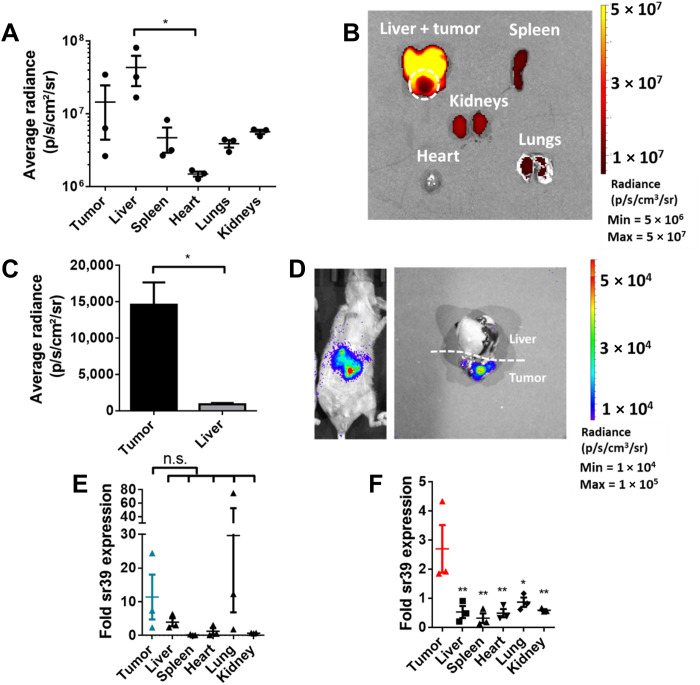
Intravenously administered NPs efficiently transfect orthotopic HCC tumors. (**A**) NP biodistribution shown as average radiance (p/s/cm^2^/sr) of major organs 1 hour after intravenous fluorescent NP administration. Significant differences between organ radiance were calculated by Kruskal-Wallis nonparametric test and Dunn’s test for multiple comparisons. *P < 0.05. Data are shown as means ± SE (*n* = 3). (**B**) Representative image of NP biodistribution 1 hour after intravenous fluorescent NP administration. (**C**) Distribution of transgene expression after intravenous administration of fLuc NPs, shown as average radiance. Differences in radiance between liver and tumor tissue were determined by a ratio-paired *t* test between average radiance over the region of interest. *P < 0.05. Data shown as means ± SE (*n* = 3). (**D**) Representative images of orthotopic tumor and liver transfected with fLuc NPs administered by intravenous injection. (**E**) Fold sr39 expression in organs after intravenous administration of CMV-sr39 NPs or (**F**) AFP-sr39 NPs by qRT-PCR. Significant differences between tumor and healthy tissue were calculated by one-way ANOVA and Dunnett’s test for multiple comparisons. *P < 0.05 and **P < 0.01, corrected for multiple comparisons. Data are shown as means ± SE (*n* = 3).

To recapitulate barriers to systemic gene delivery, we used an orthotopic xenograft model of HCC, implanting Hep3b cells in the livers of athymic NU/J mice. When PBAE 536 NP harboring fluorescently labeled DNA were administered intravenously in tumor-bearing mice, NPs largely accumulated in the liver (Fig. 6, A and B). This is in agreement with biodistribution metanalyses of NPs of this size (*46*). Of the major organs, ~7% of total fluorescence signal was localized to the tumor. To further probe gene delivery in this model, PBAE 536 NPs were used to deliver firefly luciferase (fLuc) reporter DNA in tumor-bearing mice (Fig. 6, C and D). Twenty-four hours later, transfection was localized to the tumor, with eightfold higher radiance in the tumor than liver on average.

Next, CpGf-CMV-sr39 and CpGf-AFP-sr39 were administered to tumor-bearing mice to probe sr39 expression in vivo using these two vectors. Relative expression of sr39 was determined using qRT-PCR 24 hours after NP administration. CpGf-CMV-sr39 promoted not only sr39 expression in the tumor but also off-target expression in the liver and lung (Fig. 6E). With transcriptional targeting, CpGf-AFP-sr39 NPs resulted in highly targeted sr39 expression in the tumor alone (Fig. 6F). On average, sr39 expression was higher in animals treated with CpGf-CMV-sr39 than in those treated with CpGf-AFP-sr39, which is consistent with the in vitro analysis of relative promoter strengths.

**Fig. 7.**
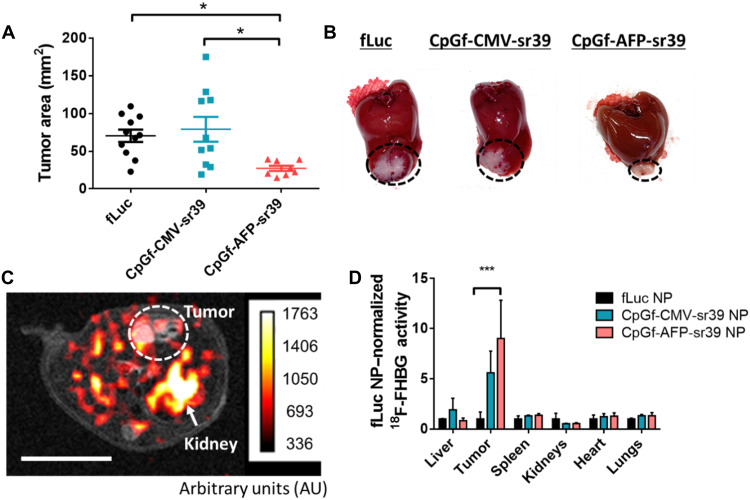
CpGf-sr39 NP treatment significantly inhibits tumor growth and enables monitoring by PET/CT. Mice with orthotopic xenograft Hep3b tumors were treated with intravenous administration of fLuc NPs (*n* = 11), CpGf-CMV-sr39 NPs (*n* = 10), or CpGf-AFP-sr39 NPs (*n* = 8) with systemic GCV. (**A**) After 16 days, tumors treated with CpGf-AFP-sr39 had significantly smaller tumors than the other two groups. Differences between treatment groups were determined by one-way ANOVA with Tukey’s posttests among the three groups. *P < 0.05. (**B**) Representative images show differences in tumor size within the liver. (**C**) Representative PET/MRI imaging shows intratumoral ^18^F-FHBG activity in a tumor-bearing mouse treated with CpGf-AFP-sr39. Scale bar, 1 cm. (**D**) Activity in major organs with fLuc (*n* = 2), CpGf-CMV-sr39 (*n* = 2), or CpGf-AFP-sr39 (*n* = 3) NPs and subsequent ^18^F-FHBG injection. Activity was calculated per mass of tissue and then normalized to activity in the control group (fLuc NP). Significant differences between treatment groups were determined by two-way ANOVA and Tukey’s posttest. ***P < 0.001. All data shown as means ± SE.

To evaluate the therapeutic potential of this approach, tumor-bearing mice were divided into treatment groups receiving intravenous injections of fLuc NPs, CpGf-CMV-sr39 NPs, or CpGf-AFP-NPs. Each animal received four NP injections spaced 4 days apart and then were euthanized 16 days after the start of treatment. All animals received GCV (50 mg/kg) daily by intraperitoneal injection. When tumors were measured at the end of the study, animals treated with CpGf-AFP-sr39 NPs had an average tumor area of 27 ± 4 mm^2^ compared with 71 ± 8 mm^2^ and 79 ± 16 mm^2^ for fLuc NP– and CpGf-CMV-sr39–treated animals, respectively (Fig. 7, A and B). This represents a significant decrease of 62% compared to fLuc NP–treated tumors. Markedly smaller tumor size is also evident in representative magnetic resonance imaging (MRI) scans of mice treated with CpGf-AFP-sr39 (fig. S4). At the end of the study, serum liver enzymes ALT and AST were found to not be elevated compared with untreated controls for any NP group (fig. S5). In addition, there were no signs of abnormalities or toxicities in tissues harvested at the end of this study, as determined by histopathology of hematoxylin and eosin–stained paraffin tissue sections, indicating safety (fig. S6).

Subsequently, this system was evaluated for in vivo PET imaging. Four weeks after tumor implantation, animals were divided into treatment groups to receive a single intravenous injection of fLuc NPs, CpGf-CMV-sr39 NPs, or CpGf-AFP-NPs. The following day, the mice received a 150-μCi injection of ^18^F-FHBG and were imaged 2 hours later with PET and T2-weighted MRI (Fig. 7C). Activity was observed in the tumor in animals treated with CpGf-AFP-sr39 (Fig. 7C), but not in animals treated with fLuc or CpGf-CMV-sr39 NPs (fig. S7). Following imaging, organs were harvested, and ^18^F activity was measured in major organs (Fig. 7D) Animals treated with CpGf-AFP-NPs showed significant and specific activity in tumor tissue. CpGf-CMV-sr39 showed lower activity in the tumor and no significant off-target transfection. This result is in agreement with the results of the therapeutic study, which unexpectedly showed no therapeutic effect from CpGf-CMV-sr39 NPs, despite strong activity in vitro. This may be due to silencing mediated by the innate immune system, which has been shown to affect strong ubiquitous viral promoters such as CMV and EF1 more strongly than tissue-specific promoters, particularly in the liver (*47*–*49*).

## DISCUSSION

Suicide gene therapy to treat cancer has shown promise but ultimately has faced several hurdles in clinical translation for patients with HCC. In a 2010 phase 1 study, a thymidine kinase–based adenoviral gene therapy was well tolerated, showing potential for safe use in humans (*50*). However, transgene expression was not detectable with repeated administrations of viral particles, indicating rapid immune recognition and silencing, leading to a modest overall median survival time of 5 months (*50*). This highlights the need for alternative delivery strategies with lower immunogenicity and improved targeting. Here, we advanced such a strategy using (i) a safe, nonviral biodegradable delivery vehicle that preferentially transfects HCC, (ii) an AFP promoter for transcriptional targeting of HCC, and (iii) completely CpG-free plasmids to reduce TLR9 activation.

PBAE NPs offer several advantages for DNA delivery to solid tumors. A large library of PBAE polymers may be synthesized rapidly using combinatorial chemistry to formulate nanomaterials with wide structural diversity and tunability (*12*). Small changes in properties including hydrophobicity, molecular weight, and effective p*K*_a_ can markedly affect cellular uptake and transfection efficacy of NPs, including cell-specific transfection (*51*–*53*). In addition, next-generation PBAE polymers incorporating bioreducible, branched, and carboxylated structures have enabled delivery of small interfering RNA (siRNA), microRNA (miRNA), and protein therapeutics (*54*–*56*). PBAE NPs have low toxicity and minimal risk of insertional mutagenesis, which are clear benefits over viral delivery strategies.

This study is the first use of PBAE NPs for systemic DNA delivery to reach liver tumors, and this was achieved without the use of a targeting ligand to a cancer cell receptor. While the targeting mechanism for PBAE 536 NPs is not fully understood, previous studies indicate that this is not due to NP size, rate of uptake, or cell division alone (*37*, *57*). We have found that NPs with clathrin-dependent uptake show improved transfection over NPs taken up by micropinocytosis or caveolae-mediated endocytosis, offering a rationale for differences in transfection rate (*58*). Still, it is evident that this targeting mechanism is complex and may be based on a combination of factors, including small differences in NP size, surface charge, nucleic acid packing, and serum protein adsorption. This nanomedicine approach for treating orthotopic tumors following intravenous injection is more clinically relevant than intratumoral injection and may be promising for treating metastases. Despite modest levels of NP accumulation within tumor tissue, PBAE 536 enabled expression of a reporter gene as well as theranostic sr39 genes in orthotopic HCC tumors with a high degree of specificity, which ultimately resulted in effective tumor cell killing and tumor imaging.

This specificity is further enhanced by incorporating the AFP promoter and enhancer for transcriptional targeting, safeguarding against systemic toxicity from systemic administration. In this study, the AFP promoter and enhancer restricted sr39 expression to AFP-producing HCC cell lines, with no off-target cell killing or radiotracer accumulation in non-HCC cells, including healthy human hepatocytes in vitro. This specificity also translated in vivo, where systemically administered CpGf-AFP-sr39 NPs transfected the tumor but not liver or other healthy mouse tissues. Overall, this clearly demonstrates the specificity of this platform for AFP-producing HCC cells. Interestingly, despite reduced promoter strength, CpGf-AFP-sr39 showed improved therapeutic activity over CpGf-CMV-sr39 in vivo. This may be due to transcriptional silencing of the strong ubiquitous CMV/EF1 promoter (*47*–*49*). Alternatively, the nontargeted platform may result in off-target transfection and killing of Kupffer cells, altering the liver and tumor microenvironments and potentially affecting tumor growth (*59*). While CpGf-AFP-sr39 NPs showed high potency in vivo, improving the strength of this promoter should be investigated to maximize therapeutic potential. Using a two-step transcriptional amplification strategy with the AFP promoter may enhance expression while maintaining cancer specificity (*19*, *60*).

A novel CpG-free version of the sr39 gene was developed and used in two theranostic plasmids: CpGf-CMV-sr39 and CpGf-AFP-sr39. Methylated CpG dinucleotides have been shown to stimulate cytokine production and reduce the duration of transgene expression (*32*). This is the first reported use of a completely CpG-free sr39 gene and AFP promoter, which we showed reduces TLR9 activation while maintaining cell killing and molecular genetic imaging functionality in vitro and in vivo, despite extensive sequence alterations. We noted that removing CpG dinucleotides from the AFP promoter and enhancer sequences resulted in a reduction of sr39 expression and activity. This highlights the importance of balancing the safety concerns of CpG sequences and promoter strength for therapeutic gene expression. Nonetheless, CpGf-AFP-sr39 showed impressive therapeutic efficacy in vivo, with a 62% reduction in tumor size compared with controls. Studies have also shown T cell involvement in thymidine kinase gene therapy (*61*), raising the possibility for further enhanced therapeutic benefit in a syngeneic model.

Overall, this approach improves upon clinically tested adenoviral TK-gene therapy by (i) using a safe, biodegradable, and flexible nonviral polymeric DNA delivery system rather than immunogenic viral vectors; (ii) removing CpG sequences to reduce TLR9 activation, which has been linked to gene silencing and inflammation in vivo; (iii) using sr39, a mutant form of HSV-TK with higher affinity for GCV and improved efficacy; and (iv) using a two-stage targeting strategy by engineering a biodegradable polymeric NP for preferential HCC delivery and using an AFP promoter for highly specific expression in HCC cells. Together, these advancements improve the clinical potential of cancer gene therapy for both imaging and therapy and may lead to improved treatment options for patients suffering from HCC.

## MATERIALS AND METHODS

### PBAE 536 synthesis

1,5-Pentanediol diacrylate (B5) (Monomer-Polymer and Dajac Labs, Trevose, PA) and 3-amino-1-propanol (S3) (Alfa Aesar, Ward Hill, MA) were combined neat at a 1:1.1 ratio for a 1.5-g total mass and allowed to polymerize under stirring for 24 hours at 90°C. This polymer was then dissolved in anhydrous tetrahydrofuran. 2-(3-Aminopropylamino)ethanol (E6) (Sigma-Aldrich, St. Louis, MO) was added in 10-fold molar excess and stirred at room temperature (RT) for 1 hour. The resulting polymer was washed two times in diethyl ether and dried under vacuum for several days. The polymer was dissolved in anhydrous DMSO and stored with desiccant at −20°C at 100 mg/ml. Molecular weight was measured by GPC relative to polystyrene standards (Waters 2414 Refractive Index Detector, Milford, MA).

### NP formation and characterization

Plasmid DNA and PBAE 536 polymer were diluted separately in sodium acetate (25 mM, pH 5) and then combined at equal volumes for a final DNA concentration of 0.03 mg/ml and polymer:DNA weight ratio (w/w) of 25. For TEM, NPs were added to a plasma-treated, carbon-coated copper grid, stained with 0.5% uranyl acetate, and then dried for 1 hour at RT. Images were acquired using a Philips/FEI BioTwin CM120 TEM. For aqueous analysis, NPs were diluted 5× in sodium acetate and then 1:1 in PBS. A Malvern Zetasizer Nano ZS (Malvern Instruments, Malvern, UK) was used to measure hydrodynamic radius and zeta potential.

### Cell culture

Hep3b [American Type Culture Collection (ATCC) HB-8064], SK-HEP-1 (ATCC HTB-52), PC-3 (ATCC CRL-1435), and THLE-3 (ATCC CRL-11233) cells were purchased from ATCC (Manassas, VA). Huh7 was provided by P. Tran’s laboratory from the Johns Hopkins University School of Medicine. Hep3b and SK-HEP-1 cells were cultured in minimal essential medium (MEM) supplemented with 10% fetal bovine serum, 1% penicillin-streptomycin, 100 μM MEM nonessential amino acids solution, and 1 mM sodium pyruvate. Huh7 and PC-3 cells were cultured in high-glucose Dulbecco’s modified Eagle medium supplemented with 10% fetal bovine serum and 1% penicillin-streptomycin. THLE-3 cells were cultured in bronchial epithelial cell growth medium (BEBM) with the additives from the kit [BEGM Bullet Kit (CC3170), Lonza/Clonetics Corp., Walkersville, MD], except gentamycin-amphotericin and epinephrine, 10% FBS, and 1% penicillin-streptomycin. For THLE-3 culture, flasks and plates were coated with 0.01 mg/ml of human fibronectin, 0.03 mg/ml of bovine collagen type 1, and 0.01 mg/ml of bovine serum albumin dissolved in BEBM basal medium and incubated overnight at 37°C. Coating solution was aspirated before seeding. THLE-3 cells were not used beyond passage 5. Cell cultures were maintained in a humidified incubator, at 37°C, with 5% CO_2_.

### In vitro transfection

Cells were seeded in 96-well plates 1 day before transfection. Medium was changed immediately before transfection. NPs were formulated as described above at 25 (w/w) and a final DNA concentration of 0.03 mg/ml (600 ng per well) or alternate dosing as specified. After waiting 10 min for assembly, 20 μl of NPs was added per well (100 μl of media). Cells were incubated with NPs for 2 hours, and then medium was changed. Viability was measured 24 hours after transfection using the MTS assay CellTiter 96 AQueous Nonradioactive Cell Proliferation Assay (Promega, Madison, WI). Transfection efficacy was determined 48 hours after using flow cytometry for GFP expression measurement. Cells were resuspended in 1× PBS with 2% fetal bovine serum (FBS) with a 1:200 dilution of propidium iodide, and an Attune NxT flow cytometer was used to measure GFP expression. Data were analyzed using FlowJo v10 (Ashland, OR). Events were gated on FSC-H and SSC-H to identify the cell population and then on BL1-A and YL1-A to gate for GFP expression in live cells.

### CMV-sr39 and AFP-sr39

Geneious 8.0.4 (Biomatters, Auckland, New Zealand) was used throughout the plasmid design process. The 1131–base pair sequence of the wild-type herpes simplex virus (type 1/strain RH2) thymidine kinase (HSV-TK) gene, obtained from the European Nucleotide Archive, was modified to produce the sr39 mutant (*39*), in which Leu, Ile, Phe, Ala, and Leu residues are replaced with Ile, Phe, Leu, Phe, and Met in the amino acid positions 159, 160, 161, 168, and 169, respectively. The 2144–base pair composite AFP enhancer and promoter sequence was obtained from the pDRVE-AFP-hAFP plasmid (InvivoGen, San Diego, CA, catalog no. pdrive-afphafp). The sr39 gene was added to the 3′ AFP enhancer and promoter sequence, separated by the Kpn I (5′-GGTACC-3′) restriction endonuclease cutting site. An Sbf I (5′-CCTGCAGG-3′) restriction endonuclease cutting site was placed on the 5′ end, and the Nhe I (5′-GCTAGC-3′) restriction endonuclease cutting site was added to the 3′ end of the construct. This entire construct was then sent for custom synthesis by GenScript (Piscataway, NJ).

To synthesize the AFP-sr39 plasmid, 10 μg each of the gene synthesis product from GenScript and pCpGRich-mcs backbone from InvivoGen (catalog no. pcpgr-mcs) were separately digested with Sbf I–HF and Nhe I–HF (New England Biolabs, Ipswich, MA) according to the manufacturer’s instructions for 10 hours at 37°C. Digestion products were run on a 0.8% agarose gel and visualized under ultraviolet light. The bands of interest were excised from the gel, and DNA was recovered using the QIAquick Gel Extraction Kit (Qiagen, catalog no. 28704). DNA concentration from extraction products was assessed with a NanoDrop 2000 spectrophotometer (Thermo Fisher Scientific), and ligation was carried out at a 1 to 7 vector to insert volume ratio. T4 DNA Ligase and buffer [NEB (New England Biolabs), catalog no. M0202S] were mixed with DNA at 4°C, and the ligation reaction was incubated at 16°C overnight. ChemiComp GT115 *Escherichia coli*, acquired frozen from InvivoGen (catalog no.gt115-11), were transformed via heat shock using 5 μl of the ligation product. Bacteria were cultured in 450 μl of SOC outgrowth medium (NEB, catalog no. B9020S) for 1 hour at 37°C. The full 500 μl of the bacteria suspension was streaked in an LB agar plate with Zeocin at 100 μg/ml. The plate was placed in a 37°C dark incubator for 16 hours. A single colony was then harvested, and bacteria were allowed to grow for additional 8 hours in LB broth (Quality Biological, catalog no.340-004-101). QIAprep Spin Miniprep Kit (Qiagen, catalog no. 27104) was used to isolate plasmid DNA, which was then sent for DNA sequencing (Sanger method). This cloning process was repeated using Kpn I–HF and Nhe I–HF to synthesize CMV-sr39.

### CpGf-CMV-sr39

To eliminate all CpG dinucleotides within the sr39 gene, CpG-creating codons, i.e., containing a CpG or forming a CpG with the preceding or succeeding codons, were replaced with non–CpG-creating synonyms by following the degenerate human genetic code. The selection of the synonymous triplet substituting a CpG-creating codon was based on the Codon Usage Tabulated from GenBank and always prioritized synonyms with higher frequency of occurrence in humans. A 10-nucleotide construct containing the Sca I (5′-AGTACT-3′) restriction endonuclease cutting site and a 10-nucleotide sequence containing the Nhe I (5′-GCTAGC-3′) restriction site were designed to flank the 5′ and 3′ ends of the gene, respectively. This construct was synthesized by Integrated DNA Technologies (IDT), and a 10-nucleotide overhang containing the Apa LI (5′-GTGCAC-3′) restriction site was subsequently incorporated into the 5′ end by PCR using FP: AATTCTGTGCACAGCTTAGACCAGTACTAT and RP: TGCTTATGCTTATATGGCTAATGCTAGCTC as primers. This CpG-free sr39 insert was cloned into the pCpGfree-vitroNmcs backbone from InvivoGen (catalog no. pcpgvtn-mcsg) with the restriction enzymes Apa LI (NEB, catalog no. R0507S) and Nhe I–HF (NEB, catalog no. R3131S) as described above.

### CpGf-AFP-sr39

To remove CpG dinucleotides from the AFP enhancer and promoter, the sequence was evaluated for putative transcription factor binding sites using the TRANSFAC database (version 8.3) through the PROMO website (*43*, *44*). A 95% similarity between predicted regulatory site and transcription factor matrix was the established threshold for a hit to be reported. CpG sequences within the AFP enhancer and promoter sequences were identified (total of six) and modified according to the following strategies: (i) Only one nucleotide was replaced within each CpG dinucleotide, and their purine or pyrimidine identity was maintained, i.e., cytosines were replaced by thymidines and guanines by adenines, and (ii) the selection of cytosine or guanine for substitution was based on the distribution of regulatory sites. In the AFP promoter and enhancer, there were no cases in which both nucleotides were identified as being a part of predicted transcription factor binding sites. Next, a designed construct consisting of the CpG-free sr39 gene was added to the 3′ end of the CpG- free AFP sequence. In addition, a 1520–base pair sequence, corresponding to base pairs 4403 to 435 of the pCpGfree-vitroNmcs vector and containing the Eco RI restriction site, was added to the 5′ end of the CpG-free AFP sequence. This entire construct was then sent for custom synthesis by GenScript (Piscataway, NJ). This CpG-free AFP-sr39 insert was cloned into the pCpGfree-vitroNmcs backbone from InvivoGen (catalog no. pcpgvtn-mcsg) with the restriction enzymes Eco RI–HF (NEB, catalog no. R3101S) and Nhe I–HF (NEB, catalog no. R3131S). Cloning was performed as described above.

### TLR9 activation assay

HEK-Blue hTLR9 cells were purchased from InvivoGen (catalog no. hkb-htlr9) and cultured according to the manufacturer’s instructions. Plasmid DNA or CpG ODN (positive control) was added at a volume of 20 μl per well. Cells were prepared in HEK-Blue Detection medium, and ~80,000 cells were added to each well (180-μl volume). Cells were incubated overnight 37°C, with 5% CO_2_. Absorbance was measured at 620 nm using a Biotek Synergy 2 plate reader (Winooski, VT).

### In vitro sr39 expression PCR

Transfection of Hep3b cells with sr39 plasmids was performed as described above. The mRNA was harvested 48 hours later, reverse transcribed, and prepared for PCR using a Cells-to-CT 1-step Power SYBR Green kit from Invitrogen (catalog no. A25600) according to the manufacturer’s instructions. The optional deoxyribonuclease (DNase) step was performed to remove plasmid DNA from the samples. The following primers were designed and used for sr39 detection: FP, GCCCTTCCTGAGGACAGACAC; RP, GGAGGCTGGGAGCTCACATG. qRT-PCR was performed using the StepOnePlus Real-Time PCR System (Applied Biosystems, Foster City, CA), with the cycling parameters specified for the Cells-to-CT kit. Threshold and baseline values were standardized across all samples, and all runs to ensure accurate comparison. The comparative CT method was used to quantify relative expression levels (*62*). Barcode amplification was normalized to the housekeeping gene glyceraldehyde-3-phosphate dehydrogenase (GAPDH) to quantify NP accumulation of each PBAE with each barcode relative to the genomic DNA content. Then, this value was normalized to nonspecific background amplification in untreated cells, by subtracting the Δ*C*_T_ of amplification in untreated cells, thereby obtaining ΔΔ*C*_T_.

$${\Delta \Delta C}_{T}={\left(C_{T\;\text{sr}39}-C_{T\;\text{GAPDH}}\right)}_{\text{treated}}-{\left(C_{T\;\text{sr}39}-C_{T\;\text{GAPDH}}\right)}_{\text{untreated}}$$

### In vitro sr39 cell killing assay

GCV was purchased from InvivoGen (catalog no. sud-gcv) and reconstituted according to the manufacturer’s instructions. Transfection of Hep3b, Huh7, SK-HEP-1, PC-3, and THLE-3 cells with sr39 plasmids was performed as described above. One day after transfection, medium was prepared with GCV at the desired concentration and then added to the cells. For long-term studies, medium with GCV was replenished every 2 days. To measure viability, the MTS assay CellTiter 96 AQueous Nonradioactive Cell Proliferation Assay (Promega, Madison, WI) was used according to the manufacturer’s instructions.

For coculture experiments, THLE-3 (5 × 10^3^ cells per well) and Huh7 (P19, 5 × 10^3^ cells per well) cells were seeded either separately or together in complete THLE-3 growth medium on precoated 96-well plates. The next day, cells were transfected with CpGf-AFP-sr39 as described and then continued to be cultured in complete THLE-3 growth medium with or without GCV (1.25 μg/ml). Five days after transfection, cells were fixed using BD Phosflow Fix Buffer I (BD Biosciences, catalog no. 557870), permeabilized using cold BD Phosflow Perm Buffer III (BD Biosciences, catalog no. 558050), and stained with PE (phycoerythrin) mouse anti-human alpha fetoprotein (BD Biosciences, catalog no. 563002) at a 1:20 dilution in Pharmingen Stain Buffer (FBS) (BD Biosciences, catalog no. 554656) at RT for 1 hour. Cells were washed twice with PBS and then incubated in PBS with Hoechst 33342 (1 μg/ml) at RT for 10 min before being imaged using a Zeiss AxioObserver fluorescence microscope. Scale bar is 200 μm for all images. Another set of cells was trypsinized, stained with Zombie Violet fixable viability dye (BioLegend), and then fixed, permeabilized, and stained with PE-AFP as previously described. The cells were analyzed using an Attune NxT (Thermo Fisher Scientific).

### Assessment of native AFP expression by cells lines

Huh7, Hep3b, SK-HEP-1, PC-3, and THLE-3 cells were stained for native AFP expression using immunocytochemistry. Cells (~150,000) were fixed using BD Phosflow Fix Buffer I (BD Biosciences, catalog no. 557870) at 37°C for 10 min. After washing with BD Pharmingen Stain Buffer (FBS) (BD Bioscences, catalog no. 554656), cells were permeabilized using cold BD Phosflow Perm Buffer III (BD Biosciences, catalog no. 558050) on ice for 30 min and washed twice with stain buffer. Cells were stained with PE mouse anti-human alpha fetoprotein (BD Biosciences, catalog no. 563002) at a 1:20 dilution in stain buffer for 20 min. Cells were then washed twice with PBS and then resuspended in a buffer solution (2% FBS in 1× PBS). Stained cells were run through a HyperCyt autosampler (IntelliCyt Corporation, Albuquerque, NM) connected to a BD Accuri C6 Flow Cytometer (BD Biosciences, San Jose, CA). The collected data were analyzed using the FlowJo software v.10.1r7 (Ashland, OR) for percentage (AFP positive %) and intensity of AFP expression (geometric mean). Gating was performed using unstained samples and was adjusted to account for varying autofluorescence between cell types. Staining was performed in triplicate.

### In vitro ^18^F-FHBG uptake assay

PET radiotracer ^18^F-FHBG was radiolabeled immediately before the study (*63*–*65*). Cells receiving no treatment were used as controls. At day 2 after transfection, ^18^F-FHBG uptake studies were performed. Huh7, Hep3b, SK-HEP-1, and PC-3 cells were treated with serum-free media for 24 hours to sync cell cycles. THLE-3 was not serum starved because of the sensitivity of this cell line. One hour before treatment, serum-free media were replaced with serum-containing media. Cells were incubated with 10 μCi/ml (0.37 MBq/ml) of freshly prepared ^18^F-FHBG for 1 hour at 37°C and then washed five times with RPMI medium containing 10% serum to remove extracellular ^18^F-FHBG. RIPA buffer (50 μl 10×) was added to the cells and incubated on ice for 5 min until cells were completely lysed. Radioactivity of the cell lysate samples was measured using an automated gamma counter (LKB Wallace 1282 Compugamma CS Universal Gamma Counter). Fifteen serial dilutions of ^18^F-FHBG were used as standards to calculate radiotracer accumulation. Protein content for each sample was measured by the Pierce BCA Protein Assay Kit (Thermo Fisher Scientific, catalog no. 23225) as directed by the manufacturer. Data were recorded as radioactivity (μCi) normalized to protein mass (μg). ^18^F-FHBG uptake studies were performed in triplicate.

### Orthotopic tumor model

All in vivo procedures were approved and overseen by the Johns Hopkins Institutional Animal Care and Use Committee. Female athymic nude mice (NU/J) were purchased from the Jackson Laboratory (Bar Harbor, ME) and were implanted with orthotopic liver tumors at 6 to 8 weeks of age. Hep3b cells with or without constitutive firefly luciferase expression (fLuc+) were resuspended in a 1:1 mixture of Hanks’ balanced salt solution and Corning Matrigel Matrix High Concentration (Corning, catalog no. 354248) at 50 million cells/ml. Before implantation, animals were anesthetized with 2.5% isoflurane in oxygen, and the skin was cleaned with povidone-iodide and ethanol. An incision was made with a scalpel extending caudally from the xiphoid process. The left lateral liver lobe was visualized, and 1 million (20 μl) cells were injected under the liver capsule. Successful inoculation with cancer cells was be verified by pale, white protrusion at the point of injection. fLuc+ Hep3b tumor growth was monitored by IVIS (in vivo imaging system) (IVIS Spectrum imaging system, PerkinElmer, Waltham, MA). d-Luciferin (150 mg/kg) (Gold Biotechnology, St. Louis, MO) was administered intraperitoneally to mice, and then, imaging was performed 8 min later. Images were analyzed across regions of interest using Living Image software (PerkinElmer, Waltham, MA).

### In vivo gene delivery to orthotopic tumors

NPs for intravenous delivery were formulated by combining DNA with PBAE 536 in sodium acetate (pH 5) for a final DNA concentration of 0.25 mg/ml and 25 (w/w). NPs were incubated at RT for 10 min to allow for self-assembly before sucrose was added to a final concentration of 90 mg/ml and a final sodium acetate concentration of 25 mM. NPs were frozen at −80°C for storage and thawed immediately before use. Four weeks after tumor implantation with non–fLuc-expressing Hep3b cells, mice received a single intravenous injection of 100 μl of NPs. For biodistribution, 10% of DNA was functionalized with amine groups using Label IT Nucleic Acid Modifying Reagent (Mirus, Beltsville, MD, # MIR 3925) and then labeled with IRDye 800RS NHS Ester (LI-COR Biosciences, Lincoln, NE). One hour after NP injection, fluorescence was measured in the organs of interest using IVIS imaging. For reporter gene transfection, fLuc expression was imaged using IVIS 24 hours after NP injection, as described above.

For qRT-PCR analysis, organs were harvested 24 hours after NP injection. Tissues were homogenized in TRIzol reagent, and then, chloroform was added for phase separation. After precipitation with isopropanol and washing with 75% ethanol, the RNA was resuspended in water. Next, the samples were treated with an RNase-Free DNase set (Qiagen, Hilden, Germany) to remove plasmid DNA. Reverse transcription was performed using the High-Capacity cDNA Reverse Transcription Kit (Thermo Fisher Scientific, Waltham, MA). Complementary DNA (cDNA) was amplified using Power SYBR Green (Thermo Fisher Scientific, Waltham, MA) and a StepOnePlus Real-Time Polymerase Chain Reaction (RT-PCR) System (Applied Biosystems) with sr39 primers: FP, GCCCTTCCTGAGGACAGACAC; RP, GGAGGCTGGGAGCTCACATG. Expression was quantified using the comparative CT method normalized to GAPDH expression. Fold expression was calculated as follows

$$\text{Fold expression}=2^{-∆∆\mathrm{CT}}$$

### sr39 therapeutic efficacy study

Mice were implanted with fLuc+ Hep3b tumors, and tumor growth was monitored by IVIS beginning 2 weeks after implantation and every 4 days thereafter. NP treatment began when tumor luminescence (total flux) reached 10^9^ p/s. Animals were randomly assigned to treatment groups to receive either fLuc NPs, CpGf-CMV NPs, or CpGf-AFP NPs. NPs were formulated for intravenous injection as described above and were administered every 4 days over 16 days, for a total of four injections. GCV was administered daily at 50 mg/kg via intraperitoneal injection, for a total of 16 injections. Sixteen days after the start of treatment, animals were anesthetized and euthanized, with blood collected by cardiac puncture for liver enzyme analysis. Blood was incubated at RT to clot and then spun down to isolate the serum. Liver enzyme levels were analyzed using an Alanine Aminotransferase Activity Assay Kit and an Aspartate Aminotransferase Activity Kit from Sigma-Aldrich (St. Louis, MO). After euthanasia, tumor area was measured using calipers. Outliers were identified and removed using the ROUT (robust regression and outlier removal) method with the most stringent criteria (*Q* = 0.1%). Significant differences between groups were identified by ordinary one-way analysis of variance (ANOVA) and Tukey’s multiple comparisons test.

For histopathology, tissues were fixed in 10% formalin for 2 days, dehydrated and cleared, and then embedded in paraffin. Paraffin sections were stained using hematoxylin and eosin and then mounted and imaged using a Zeiss AxioObserver.Z2 (Oberkochen, Germany). Sections were analyzed by a trained veterinary pathologist (Dr. Kathleen L. Gabrielson).

Before MRI, animals were anesthetized with 2.5% isoflurane in oxygen and then maintained with 2% isoflurane in oxygen for the duration of imaging experiment. Their respiration was monitored during imaging using a small animal monitoring system by SA Instruments (Stony Brook, NY). Multislice T2-weighted axial images were acquired on a 9.4-T horizontal bore MRI scanner (Bruker Biospin, Billerica, MA), equipped with a 40-mm Tx/Rx radiofrequency (RF) volume coil (Bruker Biospin GmbH) using a RARE (Rapid Imaging with Refocused Echoes) sequence [echo time (TE), 10 ms, repetition time (TR) 2500 ms, field of view (FOV) of 25 mm by 25 mm, 25 slices, 1-mm slice thickness, matrix size of 200 × 200, and 6 averages]. The images were analyzed using ImageJ (fig. S4).

### sr39 PET imaging study

Mice were implanted with fLuc+ Hep3b tumors. Four weeks after implantation, animals were imaged using IVIS to determine relative tumor size and then randomly assigned to receive either fLuc NPs, CpGf-CMV NPs, or CpGf-AFP NPs. NPs were formulated for intravenous injection as described above and were by retro-orbital injection. The next day, ^18^F-FHBG was radiolabeled (*63*–*65*), and a 150-μCi dose in an isotonic 10% ethanol solution was administered to animals via tail vein injection. Before imaging, animals were anesthetized with 2.5% isoflurane in oxygen and then maintained with 2% isoflurane in oxygen for the duration of imaging experiment. Their respiration was monitored during imaging using a small animal monitoring system by SA Instruments (Stony Brook, NY). PET-MR images were acquired 2 hours after injection on a Bruker 7 T/30 MRI scanner (Bruker BioSpin, Billerica, MA, USA) with the three-ring Bruker Si 198 PET insert. Simultaneous 10-min whole-body static PET scan and a T2-weighted (T2w) two-dimensional (2D) axial scans were acquired for two mice placed side by side on the animal bed. PET reconstructions were performed using 3D maximum likelihood expectation maximization iterative image reconstruction algorithm with a pixel size of 0.5 mm and 16 iterations. A 72-mm PET optimized Tx/Rx RF coil (Bruker Biospin GmbH) centered inside the PET detector was used for the MRI acquisition. The multislice TurboRARE T2w MRI scan had a TE/TR 30/3000 ms, FOV of 20 mm by 60 mm, 20 slices, 1-mm slice thickness, matrix size of 100 × 300, and 2 averages. The multislice T2w coronal scans were also acquired using a TurboRARE sequence with an FOV of 100 mm by 70 mm, 18 slices, 1-mm slice thickness, TR/TE 3000/36 ms, matrix size of 300 × 210, and 2 averages. After imaging, animals were euthanized, and liver, tumor, spleen, kidneys, heart, and lungs were dissected, weighed, and measured using a Wizard 2 Automatic Gamma Counter (PerkinElmer, Waltham, MA). Tissue radiopharmaceutical uptake values were calculated compared with 15 μCi ^18^F-FHBG as a standard, applying decay correction.

### Statistics

GraphPad Prism (San Diego, CA) was used for all statistical analyses. The following scheme was used to indicate statistical significance: *P < 0.05, **P < 0.01, ***P < 0.001, and ****P < 0.0001.

## Notes

View/request a protocol for this paper from *Bio-protocol*.
