# Supplementary material for: Polymeric nanoparticles for dual-targeted theranostic gene delivery to hepatocellular carcinoma
Source: Sci Adv. 2022 Jul 20;8(29):eabo6406. doi: 10.1126/sciadv.abo6406 (PMC9299552; doi:10.1126/sciadv.abo6406)
Supplement: Supplementary file 1 — Figs. S1 to S7 Table S1 [file sciadv.abo6406_sm.pdf]

Science Advances
AAAS

Supplementary Materials for

Polymeric nanoparticles for dual-targeted theranostic gene delivery to hepatocellular carcinoma

Hannah J. Vaughan *et al.*

Corresponding author: Jordan J. Green, green@jhu.edu

Sci. Adv. **8**, eabo6406 (2022)
DOI: 10.1126/sciadv.abo6406

This PDF file includes:

Figs. S1 to S7
Table S1

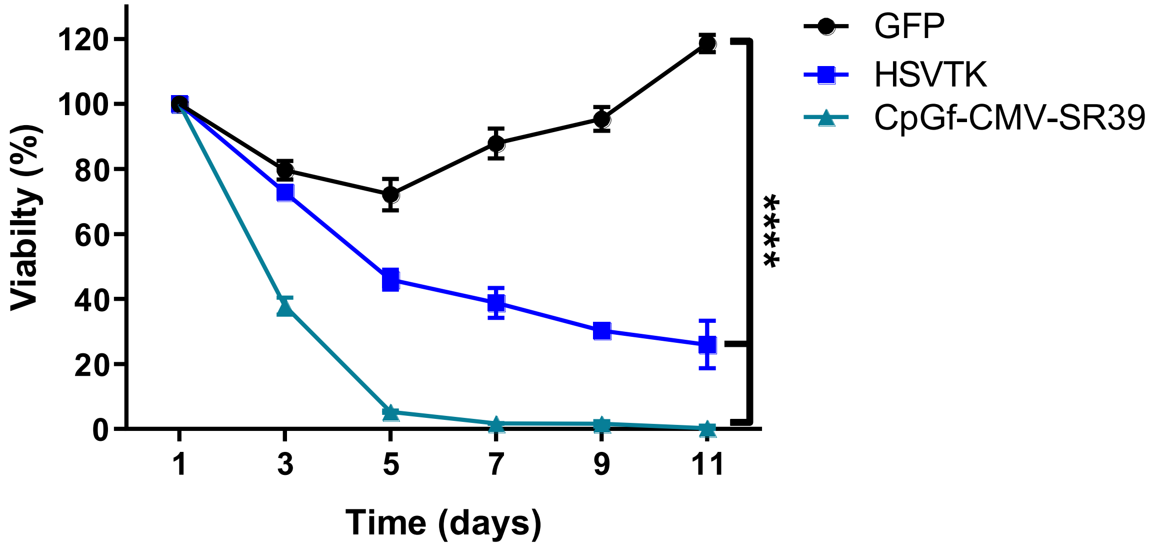

Fig. S1. CpG-free sr39 has superior cell killing effect compared with wild-type HSV-TK. Hep3b cells were transfected with NPs encapsulating GFP, HSVTK, or CpGf-CMV-sr39 DNA and treated with 1.25 µg/mL GCV on days 1, 3, 5, 7, and 9. Significant differences between groups were calculated for each time point by two-way ANOVA with Dunnett's multiple comparisons. Mean ± SE are shown (n=3) ****$P < 0.0001$.

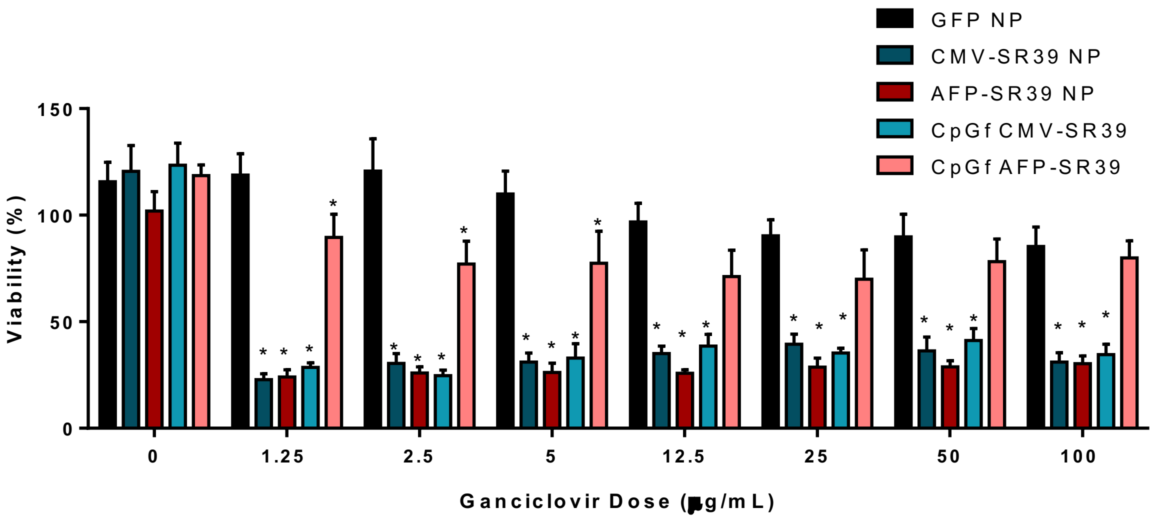

Fig. S2. sr39-mediated cell death with varying GCV doses. Hep3b cells were transfected with NPs encapsulating GFP or sr39 plasmids, then treated with GCV 1 and 3 days after transfection. Viability was measured 5 days after transfection by MTS assay. Significant differences between groups were calculated for each time point by two-way ANOVA with Dunnett's multiple comparisons. Mean ± SE are shown (n=3) *$P < 0.05$.

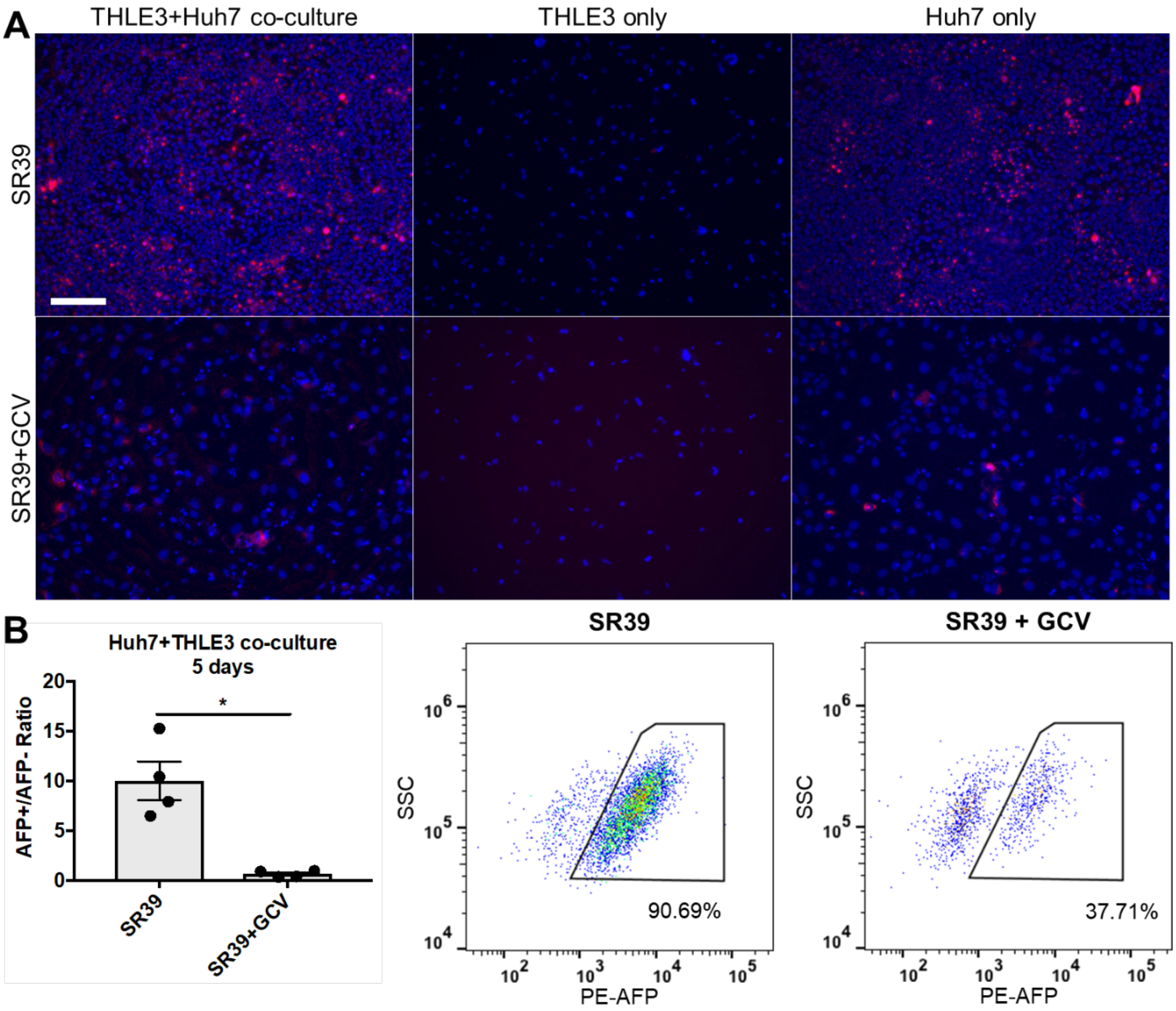

Fig. S3. HCC-specific cell death in Huh 7 and THLE-3 co-culture. Huh 7 and THLE-3 cells were cultured together or separately and transfected with CpGf-AFP-sr39. Cells were treated with 1.25 µg/mL GCV (bottom) or no GCV (top, control). After 5 days, cells were stained for AFP and (A) imaged by fluorescence microscopy or (B) evaluated by flow cytometry. Significant differences in AFP+/AFP- ratios were calculated by a two-tailed Student's t-test. Mean ± SE are shown (n=4) *P < 0.05. Scale bar = 200 µm.

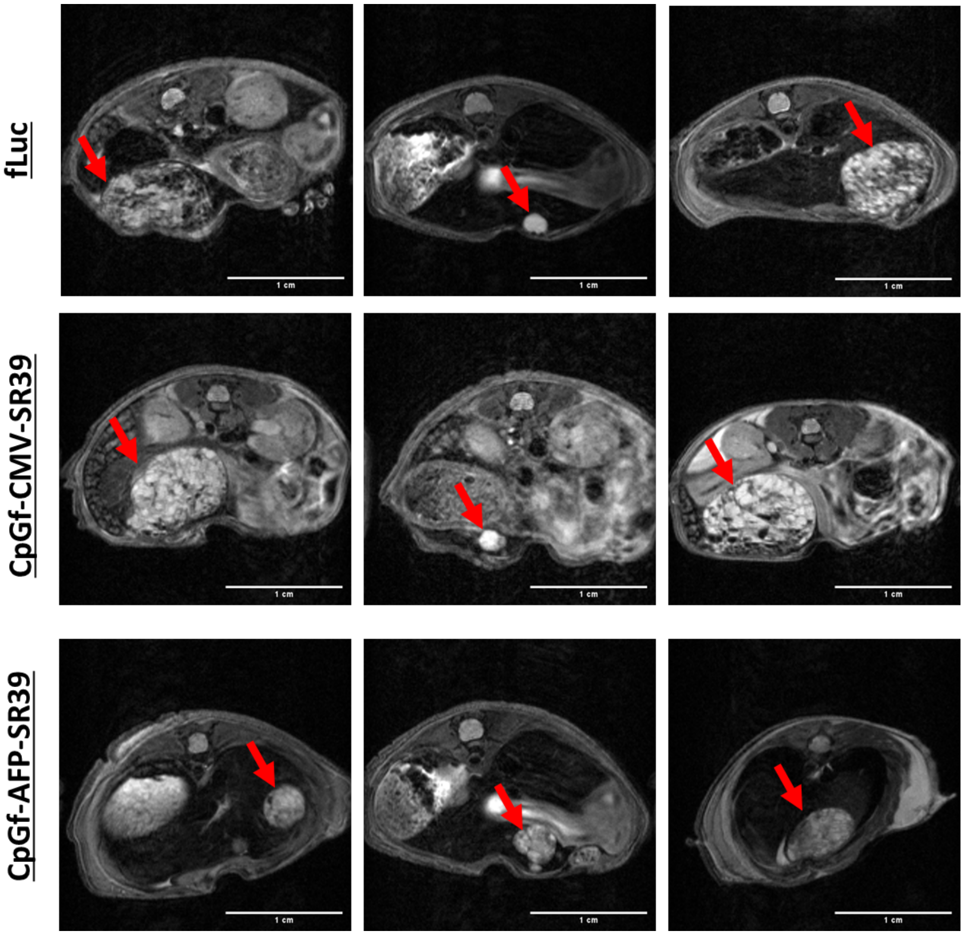

Fig. S4. T2-weighted MRI scans of the liver and HCC tumors on Day 16. Arrows point to hyperintense tumors. Scale bar = 1 cm.

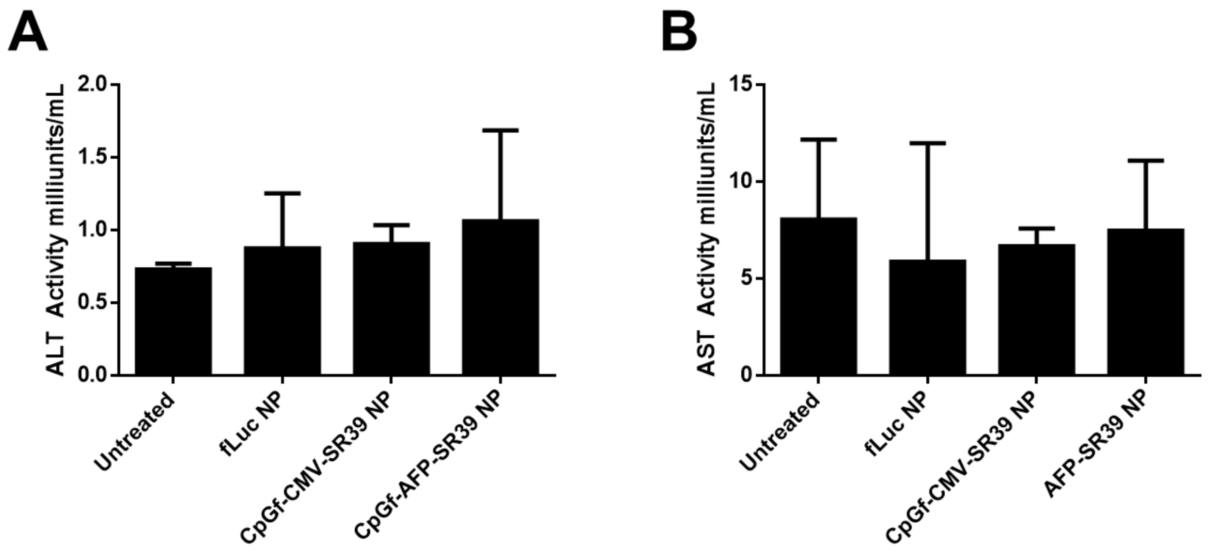

Fig. S5. Liver enzyme levels in serum after 16 days of NP + GCV treatment. (A) ALT levels and **(B)** AST levels for untreated animals (n = 3), and tumor-bearing mice treated with fLuc NPs (n = 6), CpGf-CMV-sr39 (n = 5), and CpGf-AFP-sr39 (n = 3). Mean ± SE are shown.

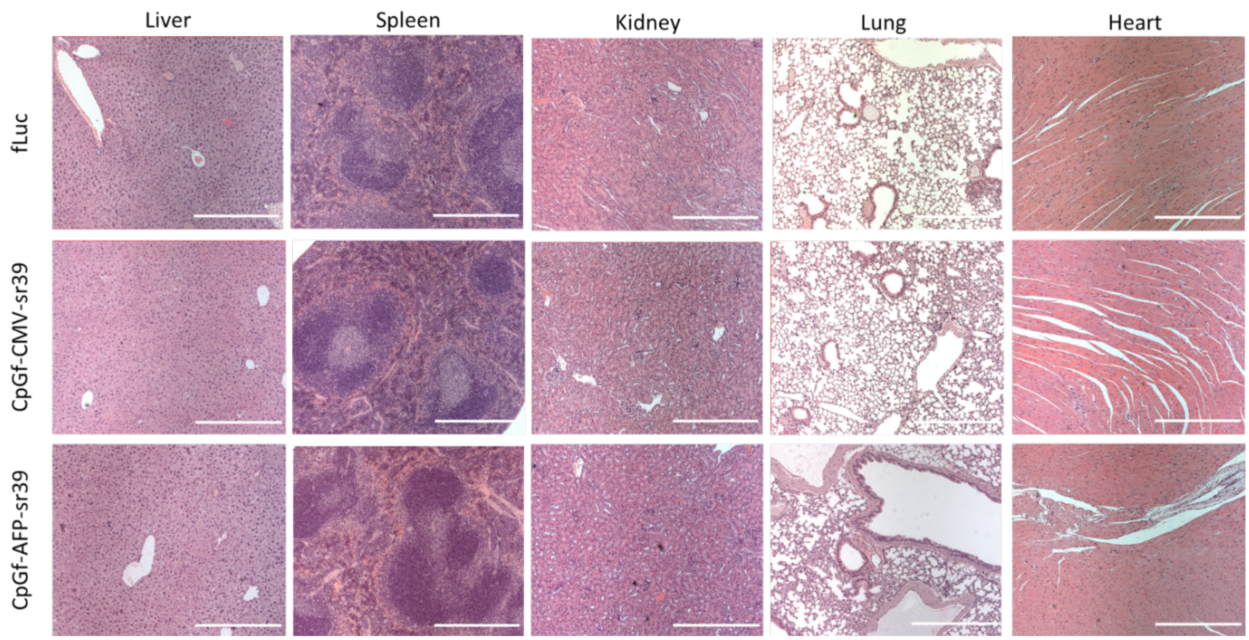

Fig. S6. Representative histopathology after 16 days of NP + GCV treatment Formalin- fixed and paraffin-embedded tissue sections from the liver, tumor, spleen, kidney, heart, and lungs. Sections were stained with hematoxylin and eosin. All images are at 10X magnification. Scale bar = 500 µm

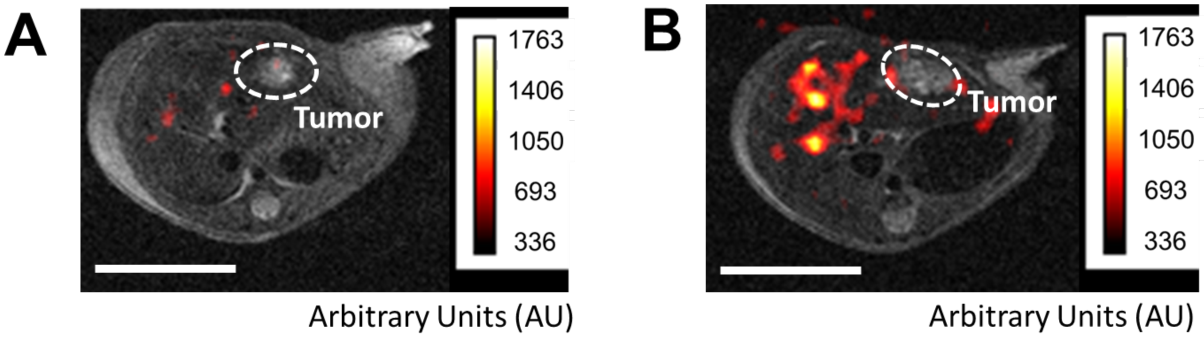

Fig. S7. Representative PET/MRI imaging of Hep3b orthotopic tumor-bearing mice. (A) Treatment with fLuc NPs and 150 mCi ^{18}F-FHBG. **(B)** Treatment with CpGf-CMV-sr39 NPs and 150 mCi ^{18}F-FHBG. Scale bar = 1 cm

Supplementary Table S1.

**CpG Free AFP Promoter/Enhancer**	GCTTAGAAATATGGGGGTAGGGGTGGTGGTGGTAATTCT GTTTTCTCCCCATAGGTGAGATAAGCATTGGGTTAAATGT GCTTTCTCTCTCTCCCTCTCCTTTCTTAAGAATTAAGGGA CAGACTATGGGCTGGAGGACTTTGAGGATGTCTGTCTCA TAACACTTGGGTTGTATCTGTTCTATGGGGCTTGTTTTAA GCTTGGCAACTTGCAACAGGGTTCACTGACTTTCTCCCC AGGCCCAAGGTACTGTCCTCTTTTCATATCTGTTTTGGGG CCTCTGGGGCTTGAATATCTGAGAAAATATAAACATTTCA ATAATGTTCTGTGGTGAGATGAGTATGAGAGATGTGTCAT TCATTTGTATCAATGAATGAATGAGGACAATTAGTGTATA AATCCTTAGTACAACAATCTGAGGGTAGGGGTGGTACTA TTCAATTTCTATTTATAAAGATACTTATTTCTATTTATTTAT GCTTGTGACAAATGTTTTGTTTGGGACCACAGGAATCACA AAGATGAGTCTTTGAATTTAAGAAGTTAATGGTCCAGGAA TAATTACATAGCTTACAAATGACTATGATATACCATCAAAC AAGAGGTTCCATGAGAAAATAATCTGAAAGGTTTAATAAG TTGTCAAAGGTGAGAGGGCTCTTCTCTAGCTAGAGACTA ATCAGAAATACATTCAGGGATAATTATTTGAATAGACCTTA AGGGTTGGGTACATTTTGTTCAAGCATTGATGGAGAAGG AGAGTGAATATTTGAAAACATTTTCAACTAACCAACCACC CAATCCAACAAACAAAAAATGAAAAGAATCTCAGAAACAG TGAGATAAGAGAAGGAATTTTCTCACAACCCACATGTATA GCTCAACTGCTCTGAAGAAGTATATATCTAATATTTAACA CTAACATCATGCTAATAATGATAATAATTACTGTCATTTTT TAATGTCTATAAGTACCAGGCATTTAGAAGATATTATTCCA TTTATATATCAAAATAAACTTGAGGGGATAGATCATTTTCA TGATATATGAGAAAAATTAAAAATCAGATTGAATTATTTGC CTGTCATACAGCTAATAATTGACCATAAGACAATTAGATTT AAATTAGTTTTGAATCTTTCTAATACCAAAGTTCAGTTTAC TGTTCCATGTTGCTTCTGAGTGGCTTCACAGACTTATGAA AAAGTAAATGGAATCAGAATTACATCAATGCAAAAGCATT GCTGTGAACTCTGTACTTAGGACTAAACTTTGAGCAATAA CACATATAGATTGAGGATTGTTTGCTGTTAGTATACAAAC TCTGGTTCAAAGCTCCTCTTTATTGCTTGTCTTGGAAAATT TGCTGTTCTTCATGGTTTCTCTTTTCACTGCTATCTATTTT TCTCAACCACTCACATGGCTACAATAACTGTCTGCAAGCT TATGATTCCCAAATGTCTATCTCTAGCCTCAATCTTGTTCC AGAAGATAAAAAGTAGTATTCAAATGCACATCAACATCTC CACTTGGAGGGCTTAAAGATGTTTCAACATACAAACTGG GGAGTTTTGCCTGGAATGTTTCCTAAAATGTGTCCTGTAG CACATAGGGTCCTCTTGTTCCTTAAAATCTAATTACTTTTA GCCCAGTGCTCATCCCACCTATGGGGAGATGAGAGTGAA AAGGGAGCCTGATTAATAATTACACTAAGTCAATAGGCAT AGAGCCAGGACTGTTTGGGTAAACTGGTCACTTTATCTTA

	AACTAAATATATCCAAAACTGAACATGTACTTAGTTACTAA GTCTTTGACTTTATCTCATTCATACCACTCAGCTTTATCCA GGCCACTAGAGTTTGAGGAGAATATTTGTTATATTTGCAA AATAAAATAAGTTTGCAAGTTTTTTTTTCTGCCCCAAAGA GCTCTGTGTCCTTGAACATAAAATACAAATAACTGCTATG CTGTTAATTATTGACAAATGTCCCATTTTCAACCTAAGGAA ATACCATAAAGTAACAGATATACCAACAAAAGGTTACTAG TTAACAGGCATTGCCTGAAAAGAGTATAAAAGAATTTCAG CATGATTTTCCATATTGTGCTTCCACCACTGCCAATAACA AAATAACTAGCAAC
**CpG Free sr39**	ATGGCTTCCTACCCTGGCCATCAGCATGCCTCTGCCTTT GACCAGGCTGCCAGATCTAGAGGCCATAGCAACAGAAGA ACTGCCTTGAGACCTAGAAGACAGCAAGAAGCCACTGAA GTCAGACCTGAGCAGAAAATGCCCACCCTACTGAGGGTT TATATAGATGGTCCCCATGGGATGGGGAAAACCACCACC ACCCAACTGCTGGTGGCCCTGGGTAGCAGAGATGATATT GTCTATGTACCTGAGCCCATGACTTACTGGAGGGTGCTG GGGGCTTCTGAGACAATTGCCAACATCTACACCACACAA CACAGACTGGACCAGGGTGAGATATCTGCTGGGGATGCT GCTGTGGTAATGACATCTGCCCAGATAACAATGGGCATG CCTTATGCTGTGACAGATGCTGTTCTGGCTCCTCATATTG GGGGGGAGGCTGGGAGCTCACATGCCCCTCCCCCTGCC CTCACCATTTTCCTGGACAGACATCCCATTGCCTTCATGC TGTGCTACCCTGCTGCCAGATACCTTATGGGCAGCATGA CCCCCCAGGCTGTGCTGGCCTTTGTGGCCCTCATCCCCC CTACCTTGCCTGGCACAAACATTGTGTTGGGGGCCCTTC CTGAGGACAGACACATTGACAGACTGGCCAAAAGACAGA GACCTGGAGAGAGACTTGACCTGGCTATGCTGGCTGCCA TTAGAAGGGTTTATGGGCTGCTTGCCAATACTGTGAGATA TCTGCAGGGAGGAGGGTCCTGGAGAGAGGATTGGGGAC AGCTTTCTGGGACTGCTGTGCCTCCCCAGGGTGCTGAGC CCCAGAGCAATGCTGGCCCAAGACCCCATATTGGGGACA CCTTATTTACCCTGTTTAGAGCCCCTGAGTTGCTGGCCC CCAATGGAGACCTGTACAATGTGTTTGCCTGGGCCTTGG ATGTCTTGGCCAAAAGACTCAGACCCATGCATGTCTTTAT CCTGGATTATGACCAATCCCCTGCTGGCTGCAGAGATGC CCTGCTGCAACTTACCTCTGGGATGGTCCAGACCCATGT CACCACCCCTGGCTCCATACCCACCATCTGTGACCTGGC CAGAACCTTTGCCAGAGAGATGGGGGAGGCTAACTGA
**AFP Promoter/Enhancer with CpG**	GCTTAGAAATATGGGGGTAGGGGTGGTGGTGGTAATTCT GTTTTCTCCCCATAGGTGAGATAAGCATTGGGTTAAATGT GCTTTCTCTCTCTCCCTCTCCTTTCTTAAGAATTAAGGGA CAGACTATGGGCTGGAGGACTTTGAGGATGTCTGTCTCA TAACACTTGGGTTGTATCTGTTCTATGGGGCTTGTTTTAA GCTTGGCAACTTGCAACAGGGTTCACTGACTTTCTCCCC AGGCCCAAGGTACTGTCCTCTTTTCATATCTGTTTTGGGG

	CCTCTGGGGCTTGAATATCTGAGAAAATATAAACATTTCA<br>ATAATGTTCTGTGGTGAGATGAGTATGAGAGATGTGTCAT<br>TCATTTGTATCAATGAATGAATGAGGACAATTAGTGTATA<br>AATCCTTAGTACAACAATCTGAGGGTAGGGGTGGTACTA<br>TTCAATTTCTATTTATAAAGATACTTATTTCTATTTATTTAT<br>GCTTGTGACAAATGTTTTGTTCGGGACCACAGGAATCAC<br>AAAGATGAGTCTTTGAATTTAAGAAGTTAATGGTCCAGGA<br>ATAATTACATAGCTTACAAATGACTATGATATACCATCAAA<br>CAAGAGGTTCCATGAGAAAATAATCTGAAAGGTTTAATAA<br>GTTGTCAAAGGTGAGAGGGCTCTTCTCTAGCTAGAGACT<br>AATCAGAAATACATTCAGGGATAATTATTTGAATAGACCTT<br>AAGGGTTGGGTACATTTTGTTCAAGCATTGATGGAGAAG<br>GAGAGTGAATATTTGAAAACATTTTCAACTAACCAACCAC<br>CCAATCCAACAAACAAAAAATGAAAAGAATCTCAGAAACA<br>GTGAGATAAGAGAAGGAATTTTCTCACAACCCACACGTAT<br>AGCTCAACTGCTCTGAAGAAGTATATATCTAATATTTAACA<br>CTAACATCATGCTAATAATGATAATAATTACTGTCATTTTT<br>TAATGTCTATAAGTACCAGGCATTTAGAAGATATTATTCCA<br>TTTATATATCAAAATAAACTTGAGGGGATAGATCATTTTCA<br>TGATATATGAGAAAAATTAAAAATCAGATTGAATTATTTGC<br>CTGTCATACAGCTAATAATTGACCATAAGACAATTAGATTT<br>AAATTAGTTTTGAATCTTTCTAATACCAAAGTTCAGTTTAC<br>TGTTCCATGTTGCTTCTGAGTGGCTTCACAGACTTATGAA<br>AAAGTAAACGGAATCAGAATTACATCAATGCAAAAGCATT<br>GCTGTGAACTCTGTACTTAGGACTAAACTTTGAGCAATAA<br>CACATATAGATTGAGGATTGTTTGCTGTTAGTATACAAAC<br>TCTGGTTCAAAGCTCCTCTTTATTGCTTGTCTTGGAAAATT<br>TGCTGTTCTTCATGGTTTCTCTTTTCACTGCTATCTATTTT<br>TCTCAACCACTCACATGGCTACAATAACTGTCTGCAAGCT<br>TATGATTCCCAAATGTCTATCTCTAGCCTCAATCTTGTTCC<br>AGAAGATAAAAAGTAGTATTCAAATGCACATCAACGTCTC<br>CACTTGGAGGGCTTAAAGACGTTTCAACATACAAACCGG<br>GGAGTTTTGCCTGGAATGTTTCCTAAAATGTGTCCTGTAG<br>CACATAGGGTCCTCTTGTTCCTTAAAATCTAATTACTTTTA<br>GCCCAGTGCTCATCCCACCTATGGGGAGATGAGAGTGAA<br>AAGGGAGCCTGATTAATAATTACACTAAGTCAATAGGCAT<br>AGAGCCAGGACTGTTTGGGTAAACTGGTCACTTTATCTTA<br>AACTAAATATATCCAAAACTGAACATGTACTTAGTTACTAA<br>GTCTTTGACTTTATCTCATTCATACCACTCAGCTTTATCCA<br>GGCCACTAGAGTTTGAGGAGAATATTTGTTATATTTGCAA<br>AATAAAATAAGTTTGCAAGTTTTTTTTTTCTGCCCCAAAGA<br>GCTCTGTGTCCTTGAACATAAAATACAAATAACCGCTATG<br>CTGTTAATTATTGACAAATGTCCCATTTTCAACCTAAGGAA<br>ATACCATAAAGTAACAGATATACCAACAAAAGGTTACTAG<br>TTAACAGGCATTGCCTGAAAAGAGTATAAAAGAATTTCAG

	CATGATTTTCCATATTGTGCTTCCACCACTGCCAATAACA AAATAACTAGCAAC
**sr39 with CpG**	ATGGCTTCGTACCCCGGCCATCAGCACGCGTCTGCGTTC GACCAGGCTGCGCGTTCTCGCGGCCATAGCAACCGACG TACGGCGTTGCGCCCTCGCCGGCAGCAAGAAGCCACGG AAGTCCGCCCGGAGCAGAAAATGCCCACGCTACTGCGG GTTTATATAGACGGTCCCCACGGGATGGGGAAAACCACC ACCACGCAACTGCTGGTGGCCCTGGGTTCGCGCGACGA TATCGTCTACGTACCCGAGCCGATGACTTACTGGCGGGT GCTGGGGGCTTCCGAGACAATCGCGAACATCTACACCAC ACAACACCGCCTCGACCAGGGTGAGATATCGGCCGGGG ACGCGGCGGTGGTAATGACAAGCGCCCAGATAACAATG GGCATGCCTTATGCCGTGACCGACGCCGTTCTGGCTCCT CATATCGGGGGGGAGGCTGGGAGCTCACATGCCCCGCC CCCGGCCCTCACCATTTTCCTGGACCGCCATCCCATCGC CTTCATGCTGTGCTACCCGGCCGCGCGATACCTTATGGG CAGCATGACCCCCCAGGCCGTGCTGGCGTTCGTGGCCC TCATCCCGCCGACCTTGCCCGGCACAAACATCGTGTTGG GGGCCCTTCCGGAGGACAGACACATCGACCGCCTGGCC AAACGCCAGCGCCCCGGCGAGCGGCTTGACCTGGCTAT GCTGGCCGCGATTCGCCGCGTTTACGGGCTGCTTGCCA ATACGGTGCGGTATCTGCAGGGCGGCGGGTCGTGGCGG GAGGATTGGGGACAGCTTTCGGGGACGGCCGTGCCGCC CCAGGGTGCCGAGCCCCAGAGCAACGCGGGCCCACGA CCCCATATCGGGGACACGTTATTTACCCTGTTTCGGGCC CCCGAGTTGCTGGCCCCCAACGGCGACCTGTACAACGT GTTTGCCTGGGCCTTGGACGTCTTGGCCAAACGCCTCCG TCCCATGCACGTCTTTATCCTGGATTACGACCAATCGCCC GCCGGCTGCCGGGACGCCCTGCTGCAACTTACCTCCGG GATGGTCCAGACCCACGTCACCACCCCCGGCTCCATACC GACGATCTGCGACCTGGCGCGCACGTTTGCCCGGGAGA TGGGGGAGGCTAACTGA

Table S1. Sequences for sr39 plasmid components
